# The influence of hospital type (public versus private) on mortality and survival after stroke

**DOI:** 10.1055/s-0045-1809418

**Published:** 2025-06-20

**Authors:** Carla Heloisa Cabral Moro, Maria Izabel Rodrigues Mendes, Milena Veiga Wiggers, Thaís de Faria Cardoso, Henrique Diegoli, Luciano Henrique Pinto, Helbert do Nascimento Lima

**Affiliations:** 1 Hospital Municipal São José, Departamento de Neurologia, Joinville SC, Brazil.; 2 Universidade da Região de Joinville, Faculdade de Medicina, Joinville SC, Brazil.; 3 Academia VBHC, São Paulo SP, Brazil.; 4 Universidade da Região de Joinville, Programa de Pós-Graduação em Saúde e Meio Ambiente, Joinville SC, Brazil.

**Keywords:** Stroke, Survival Analysis, Health Care Economics and Organizations

## Abstract

**Background:**

Stroke has been a leading cause of death in Brazil throughout the past three decades. Although several cities in the country have implemented urgent/emergency stroke care units, the impact of the hospital type (public versus private) has not been evaluated.

**Objective:**

To compare the mortality and survival of patients admitted with stroke to two private hospitals without stroke units with a public hospital with a stroke unit.

**Methods:**

We conducted a historical cohort in the city of Joinville, Southern Brazil. Stroke patients admitted to a public hospital with a stroke unit were compared with those admitted to private hospitals without stroke units in terms of fatality rate and 30-day survival between January 2018 and December 2020. The Cox regression was used.

**Results:**

Of the 4,508 patients, 85.6% were from public hospital, and 14.4%, from the 2 private hospitals. The crude mortality rate was of 11.4% among the public hospital patients, and of 9.1% among the patients from the private hospitals ( *p* = 0.085). In the multivariate analysis, there was no difference in mortality between patients treated in the public hospital with a stroke unit (hazard ratio = 1.21; 95%CI: 0.87–1.68; *p* = 0.262) and those admitted to the 2 private hospitals without a stroke unit.

**Conclusion:**

Stroke units are an important public policy that minimizes the impact of stroke.

## INTRODUCTION

Stroke has been a leading cause of death in Brazil throughout the past three decades. 1 Although the crude mortality rate from stroke has been decreasing, more than 63 thousand people had lost their lives to stroke in the country by August 2024. 1 Several cities in Brazil have implemented urgent/emergency stroke care in public and private healthcare networks. 2 However, few studies have evaluated the characteristics and survival of stroke patients according to the type of hospital in which they received care: public or private.

Currently, health spending in Brazil represents ∼ 9% of the Gross Domestic Product, 56% of which comes from the private health system, and 44%, from the public system. 3 The Brazilian Unified Health System (Sistema Único de Saúde, SUS, in Portuguese) is responsible for serving the entire Brazilian population free of charge, with 75% of Brazilians relying exclusively on it. 3 In 2020, Federação Brasileira de Hospitais (Brazilian Federation of Hospitals) and Confederação Nacional de Saúde (National Health Confederation) 4 reported that the number of public hospitals in the country would increase slightly between 2010 and 2022. However, the recommended number of beds per one thousand inhabitants nationwide (2.3) is still below the recommendation of the World Health Organization recommendation (3.0), and the ratio rises to 3.5 beds per one thousand inhabitants when beds in the privately-insured population are considered. 5 Considering that most stroke patients are treated in hospitals, those that are not prepared to care for these patients may experience an impact on the clinical outcomes resulting from the disease and its complications. 6 Since 2012, the Brazilian Ministry of Health has encouraged the creation of stroke units (through Ordinance no. 665/2012). These units would have a dedicated multidisciplinary area for acute and subacute care, computed tomography (CT) scans, an intensive care unit, and a team of specialists in neurology and neurosurgery available 24 hours a day. Studies 6 7 suggest hospitals with dedicated stroke units can provide more effective care and minimize outcomes, especially mortality. However, studies conducted in Brazil are still limited.

Given the high incidence of stroke in the country and the implementation of care networks for the acute care of these patients, the present study aimed to compare the characteristics, mortality, and survival of patients admitted to two private hospitals without stroke units and of those admitted to a public hospital with a stroke unit in the city of Joinville, state of Santa Catarina, Southern Brazil.

## METHODS

### Design, site, and sampling

The current is a historical cohort study based on data from the JOINVASC epidemiological stroke registry, which is derived from a population-based cohort in Joinville that has been ongoing since 1995 and monitors all in-hospital and out-of-hospital stroke cases in the city. 8 The methodology used for data collection in JOINVASC follows the STEPS modular program recommended by the World Health Organization for the evaluation of stroke. 9 Joinville is the third most populous city in southern Brazil, with an estimated population of ∼ 616 thousand according to the last census. 10 The city has five general hospitals, three private and two public. The data analyzed were from January 2018 to December 2022. All hospitalized patients diagnosed with stroke (ischemic, hemorrhagic, or transient ischemic attack) of both sexes and aged 18 years or older were included. We considered the hospitals in the city with the highest number of stroke cases, Centro Hospitalar Unimed (CHU) and Hospital Dona Helena (HDH), both private, and Hospital Municipal São José (HMSJ), a public hospital that receives all stroke cases from the public health network of Joinville, with a specific multidisciplinary inpatient unit for stroke. Participants without complete data available in JOINVASC and those with other types of cerebral events (subarachnoid hemorrhage and venous sinus thrombosis) were excluded. This study was approved by the HMSJ Human Research Ethics Committee (under CAAE 69867523.1.0000.5366).

### Variables collected and tools used

We collected the following data from JOINVASC: sociodemographic characteristics (age, sex, ethnicity), date of admission, date of discharge,, history of comorbidities (diabetes, ischemic heart disease [acute myocardial infarction, angioplasty, or myocardial revascularization surgery], dyslipidemia), body mass index (BMI), type of stroke, atrial fibrillation, severity of stroke according to the National Institutes of Health Stroke Scale (NIHSS), and performance of reperfusion therapy (thrombectomy and/or thrombolytic therapy). The laboratory levels of glucose and creatinine were also collected on hospital admission. The level of creatinine on admission was used to estimate the glomerular filtration rate using the Chronic Kidney Disease Epidemiology Collaboration (CKD-EPI) formula. 11 The admission periods of each stroke patient were considered in relation to the coronavirus disease 2019 (COVID-19) pandemic: prepandemic (from January 2018 to February 2020), pandemic period (from March 2020 to September 2021), and postpandemic (from October 2021 to December 2022). The outcome studied was in-hospital death within 30 days of admission. The participants were divided into public care (HMSJ) and private care (HDH or CHU).

### Statistical analysis

Descriptive statistics were used to express the baseline characteristics of the entire cohort. Variables defined as categorical data are expressed as frequencies and proportions, and numerical variables, as median and interquartile range (IQR) values. The distribution of the variables is presented by outcome (death from any cause up to 30 days after hospitalization for stroke) and by primary exposure (hospital type: public versus private). For patients discharged before 30 days since the admission, the outcome studied (death) was followed up through telephone contact until 30 days after discharge. We used the Chi-squared test to assess differences in distribution among the categorical variables and the Mann-Whitney test for the numerical variables, after testing for normality with the Kolmogorov-Smirnov test. The Kaplan-Meier method was used to analyze survival curves according to hospital type, and the logrank test was used to assess any differences. A crude univariate analysis was performed to verify the association between the primary variable and death using Cox regression. The proportional hazards assumption of the Cox regression was tested graphically. The effect of the primary exposure was then adjusted through bivariate analysis for the other potentially-confounding variables. All variables with a known confounding effect or an effect modification for the exposure studied greater or lower than 5% in the bivariate analysis were included in the final multivariate model through Cox regression analysis. Using the likelihood ratio test (LRT), we tested for an interaction effect between the type of hospital and the aforementioned admission periods. We tested the need for multilevel analysis using the intraclass correlation coefficient (ICC) among hospitals. The ICC found (0.002; 95%CI: 0.0001–0.03) did not suggest the presence of data dependence and the need for multilevel analysis. Values of *p* < 0.05 were considered significant. All data were analyzed using Stata/IC 15.1 (StataCorp. LLC) software.

## RESULTS

Of the total sample of 4,742 patients, 161 (3.4%) were excluded because they had other types of stroke (157 with subarachnoid hemorrhage and 4 with venous sinus thrombosis). Of the 4,508 patients analyzed, 3,923 (85.6%) were treated in the public hospital and 658 (14.4%), in the two private hospitals. The overall mortality rate was of 11.1% (public: 11.4%; private: 9.1%; *p* = 0.085). Table 1 shows the general characteristics of the patients stratified by hospital type. Regarding the age of the patients, 64.8% were older than 65 years, and 53.5% were male subjects. Systemic arterial hypertension was the most common comorbidity, found in 78.4% of the patients, and 10.5% presented a reduction in the estimated glomerular filtration rate below 45 mL/minute/1.73m ^3^ . Regarding the period of admission to the hospitals analyzed, 41.8% of the admissions were prepandemic, 30.1%, pandemic, and 28.1%, postpandemic. Reperfusion therapy was performed in 10.7% of the sample: thrombolytic therapy in 64%, thrombectomy in 25%, and both in 11%. Among the types of stroke, the ischemic type was the most common (77.6%). The 30-day mortality rate was 11.1%. Of the 507 deaths, 446 (88%) were due to a new stroke, 22 (4.3%), due to sepsis, 8 (1.6%), due to acute cardiac events, 8 (1.6%), due to cancer complications, and 26 (5.1%), due to other causes. When the sample was stratified according to the type of hospital, the public hospital group presented a lower proportion of patients older than 75 years, a higher proportion of male subjects, a lower proportion of Caucasian individuals, a higher proportion of patients with hypertension and diabetes, and a higher incidence of severe cases (NIHSS > 10; 62.1%; *p* < 0.001). There was no difference in terms of the performance of reperfusion therapy according to hospital type. Regarding the admission periods, the public hospital admitted a higher number of patients with stroke during the pandemic compared with the 2 private hospitals (30.9% versus 25.2%; *p* = 0.013).

**Table 1:** General characteristics of the study sample stratified by type of hospital

Variables		Total sample (N = 4,581)		Public (n = 3,923; 85.6%)		Private (n = 658; 14.4%)		*p* -value
**Age group** (years): n (%)	18–45	225	(5.4%)	179	(4.6%)	46	(6.7%)	< 0.001
	45–65	1,412	(29.8%)	1,152	(29.4%)	184	(28.0%)	
	66–75	1,423	(30.0%)	1,230	(31.3%)	159	(24.2%)	
	> 75	1,651	(34.8%)	1,362	(34.7%)	269	(40.9%)	
**Male sex** : n (%)		2,452	(53.5%)	2,135	(54.4%)	317	(49.2%)	0.003
**Color or race** : n (%)	White	4,243	(92.6%)	3,619	(92.2%)	624	(94.8%)	0.019
	Non-white	338	(7.4%)	304	(7.7%)	34	(5.2%)	
**BMI** (kg/m ^2^ ): median (IQR)		26.8	23.9–30.1	26.8	24–30.1	26.6	23.8–19.9	0.355
**Hospital type** : n (%)	Public	3,923	(85.6%)					
	Private	658	(14.4%)					
**Admission period** : n (%)	01/2018–02/2020	1,916	(41.8%)	1,618	(41.2%)	298	(45.3%)	0.013
	03/2020–09/2021	1,378	(30.1%)	1,212	(30.9%)	166	(25.2%)	
	10/2021–12/2022	1,287	(28.1%)	1,093	(27.9%)	194	(29.5%)	
**Type of stroke** : n (%)	TIA	663	(14.5%)	558	(14.2%)	105	(16.0%)	0.372
	Ischemic	3,557	(77.6%)	3,050	(77.7%)	507	(77.0%)	
	Hemorrhagic	361	(7.9%)	315	(8.0%)	46	(7.0%)	
**Reperfusion therapy** (yes): n (%)		489	(10.7%)	405	(10.3%)	84	(12.8%)	0.060
**NIHSS score** (N = 1,574): n (%)	< 4	2,419	(53.4%)	2,038	(52.0%)	381	(62.1%)	< 0.001
	4–10	1,186	(26.2%)	1,069	(27.3%)	117	(19.1%)	
	>10	928	(20.5%)	813	(20.7%)	115	(18.8%)	
**Previous IHD** (yes): n (%)		455	(9.9%)	367	(9.4%)	88	(13.4%)	0.001
**Hypertension** (yes): n (%)		3590	(78.4%)	3108	(79.2%)	482	(73.2%)	0.001
**Diabetes** (yes): n (%)		1,712	(37.4%)	1,497	(38.2%)	215	(32.7%)	0.007
**Dyslipidemia** (yes): n (%)		1,843	(40.2%)	1,546	(39.4%)	297	(45.1%)	0.006
**Glucose** (mg/dL; N = 4,578): median (IQR)		114	97–151	114	97–151	114	98–151	0.708
**eGFR** (mL/minute/1.73m ^2^ ; N = 4,531): n (%)	≥ 60	3,384	(77.3%)	2,927	(77.8%)	457	(74.1%)	0.014
	45–59	532	(12.1%)	435	(11.6%)	97	(15.7%)	
	< 45	461	(10.5%)	398	(10.6%)	63	(10.2%)	
**Atrial fibrillation** (yes): n (%)		397	(8.7%)	332	(8.5%)	65	(9.9%)	0.232
**Length of hospital stay** (days): median (IQR)		8	4–13	8	5–14	5	3–9	< 0.001
**Death within 30 days** (yes): n (%)		507	(11.1%)	447	(11.4%)	60	(9.1%)	0.085

Abbreviations: BMI, body mass index; eGFR, estimated glomerular filtration rate; IHD, ischemic heart disease (myocardial infarction, coronary artery bypass graft, coronary angioplasty); IQR, interquartile range (25th percentile–75th percentile); NIHSS, National Institutes of Health Stroke Scale; TIA, transient ischemic attack.

Table 2 shows the clinical characteristics of the sample concerning whether they died during the first 30 days after admission. Compared with the survivors, patients who died presented a higher prevalence of age > 75 years, white race, hemorrhagic stroke, and greater severity on the NIHSS (score > 10 points; 70.2% versus 14.3%; *p* < 0.001). The presence of atrial fibrillation, dyslipidemia, higher blood glucose levels on admission, chronic kidney disease, as well as the admission periods were different among patients who died within 30 days of admission.

**Table 2:** General characteristics according to survival during hospital admission

Variable		Survived (n = 4,074; 88.9%)		Died (n = 507; 11.1%)		*p* -value
**Age group** (years): n (%)	18–45	213	(5.2%)	12	(2.4%)	< 0.001
	45–65	1,244	(30.5%)	92	(18.1%)	
	66–75	1,268	(31.1%	121	(23.9%)	
	> 75	1,249	(33.1%)	282	(55.6%)	
**Male sex** : n (%)		225	(54.6%)	227	(44.8%)	< 0.001
**Color or race** : n (%)	White	3,760	(92.3%)	483	(95.3%)	0.016
	Non-white	314	(7.7%)	24	(4.7%)	
**BMI** (kg/m ^2^ ): median (IQR)		26.9	24.1–30.1	25.8	23.1–29.4	< 0.001
**Hospital type** : n (%)	Public	3,476	(85.3%)	447	(88.2%)	0.085
	Private	598	(14.7%)	60	(11.8%)	
**Admission period** : n (%)	01/2018–02/2020	1,719	(42.2%)	197	(38.9%)	0.033
	03/2020–09/2021	1,200	(29.5%)	178	(35.1%)	
	10/2021–12/2022	1,155	28.3%)	132	(26.0%)	
**Type of stroke** : n (%)	TIA	659	(16.2%)	4	(0.8%)	< 0.001
	Ischemic	3,187	(78.2%)	370	(73.0%)	
	Hemorrhagic	228	(5.6%)	133	(26.2%)	
**Reperfusion therapy** (yes): n (%)		426	(10.5%)	63	(12.4%)	0.176
**NIHSS score** (N = 1,574): n (%)	< 4	2,362	(58.6%)	57	(11.3%)	< 0.001
	4–10	1,093	(27.1%)	93	(18.5%)	
	>10	575	(14.3%)	353	(70.2%)	
**Previous IHD** (yes): n (%)		394	(9.7%)	61	(12.0%)	0.094
**Hypertension** (yes): n (%)		3,190	(78.3%)	400	(78.9%)	0.759
**Diabetes** (yes): n (%)		1,523	(37.4%)	189	(37.3%)	0.963
**Dyslipidemia** (yes): n (%)		1,611	(39.5%)	232	(45.8%)	0.007
**Glucose** (mg/dL; N = 4,578): median (IQR)		111	96–146	138	111–184	< 0.001
**eGFR** (mL/minute/1.73m ^2^ ; N = 4,531): n (%)	≥ 60	3,086	(79.4%)	298	(60.6%)	< 0.001
	45–59	452	(11.6%)	80	(16.3%)	
	< 45	347	(8.9%)	114	(23.2%)	
**Atrial fibrillation** (yes): n (%)		310	(7.6%)	87	(17.2%)	< 0.001
**Length of hospital stay** (days); median (IQR)		8	5–13	6	3–13	< 0.001

Abbreviations: BMI, body mass index; eGFR, estimated glomerular filtration rate; IHD, ischemic heart disease (myocardial infarction, coronary artery bypass graft, coronary angioplasty); IQR, interquartile range (25th percentile–75th percentile); NIHSS, National Institutes of Health Stroke Scale; TIA, transient ischemic attack.

**Figure 1:**
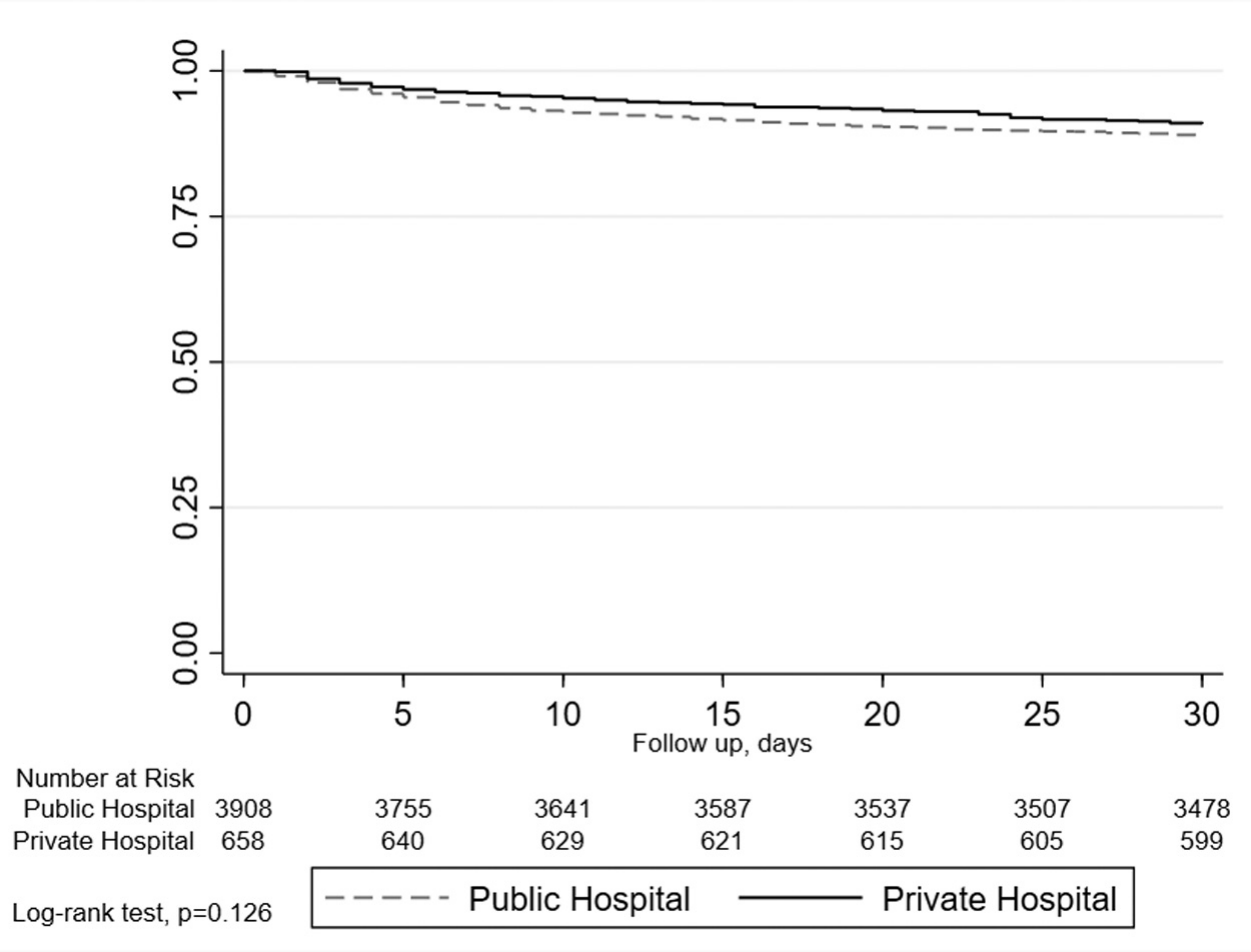
30-day survival curve after stroke using the Kaplan-Meier method.

Figure 1 shows the crude survival curve up to 30 days after the brain event. There was no difference in survival between public and private hospital patients (logrank test; *p* = 0.126). Survival at 30 days was of 90.9% (95%CI: 88.4–92.8%) for the private hospital patients and of 88.9% (95%CI: 87.9–89.9%) for the public hospital patients.

The crude probability of death during the observation period ( Table 3 ) was not different between the two groups of patients (HR = 1.23; 95% CI 0.94–1.62; *p* = 0.128). When this effect was adjusted for other confounding variables on a bivariate basis, only BMI, NIHSS, age, and admission glucose modified the effect of the hospital type variable by 5% or more, but only for admission glucose was this effect significant.

**Table 3:** Crude and adjusted univariate analysis for death within 30 days of ischemic stroke using Cox regression (N = 1,482)

	Crude HR	95%CI	*p* -value
**Type of hospital** (public versus private)	1.23	0.94–1.62	0.128
**Effect of hospital type, adjusted for:**			
**Age** (years)	1.30	0.99–1.71	0.056
**Sex** (male versus female)	1.26	0.96–1.65	0.089
**Color or race** (white versus non-white)	1.25	0.95–1.63	0.109
**BMI** (kg/m ^2^ )	1.14	0.87–1.49	0.355
**Reperfusion therapy** (yes)	1.24	0.95–1.62	0.118
** NIHSS score ^a^**	1.17	0.89–1.55	0.255
**Stroke** type ^c^	1.20	0.91–1.57	0.195
**Hypertension** (yes)	1.23	0.94–1.61	0.133
**Diabetes** (yes)	1.23	0.94–1.61	0.129
**Dyslipidemia** (yes)	1.25	0.95–1.64	0.105
**Ischemic heart disease** (yes)	1.25	0.95–1.64	0.104
**Atrial fibrillation** (yes)	1.23	0.96–1.64	0.102
** Admission period ^b^**	1.22	0.93–1.59	0.155
**Glucose** (mg/dl)	1.39	1.02–1.90	0.037
**eGFR** (mL/minute/1.73m ^2^ )	1.21	0.92–1.59	0.163

Abbreviations: BMI, body mass index; eGFR, estimated glomerular filtration rate; HR, hazard ratio; NIHSS, National Institutes of Health Stroke Scale.

Notes: ^a^ Assuming a linear effect and the first category as a reference (NIHSS score < 4). ^b^ Assuming a linear effect and the first period as a reference (01/2018–02/2020). ^c^ Assuming a linear effect for stroke type, being TIA as reference.

After adjustment for these variables and those known to confound the association between hospital type and death ( Table 4 ), hospitalization in a public hospital remained unassociated with a higher risk of death within 30 days compared with patients admitted to private hospitals (hazard ratio [HR] = 1.21; 95%CI: 0.87–1.68; *p* = 0.262). The result of the multivariate analysis did not show any interaction with the COVID-19 pandemic period (LRT = 1,000).

**Table 4:** Multivariate analysis of the effect of hospital type on death within 30 days of stroke using Cox regression

		HR	95%CI	*P* -value
**Hospital type** (public versus private)		1.20	0.86–1.67	0.275
**Agegroup** (years)	18–45	1.00	Reference	
	45–65	1.18	0.57–2.47	0.651
	66–75	1.50	0.72–3.13	0.273
	> 75	2.48	1.21–5.07	0.013
**Gender** (male versus female)		0.87	0.71–1.07	0.189
**Reperfusion therapy** (yes)		0.65	0.48–0.88	0.005
**NIHSS score**	< 4	1.00	Reference	
	4–10	3.41	2.31–5.03	< 0.001
	> 10	14.85	11.10–22.62	< 0.001
**Glucose** (mg/dL, per unit increase)		1.00	1.00–1.01	< 0.001
**BMI** (kg/m ^2^ , per unit increase)		0.97	0.95–0.99	0.007
**Stroke type**	TIA	1.00	Reference	
	Ischemic	3.74	1.36–10.27	0.010
	Hemorrhagic	9.67	3.44–27.16	< 0.001
**Ischemic heart disease** , yes		1.39	1.03–1.88	0.029
**Admission period**	01/2018–02/2020	1.00	Reference	
	03/2020–09/2021	1.15	0.92–1.45	0.215
	10/2021–12/2022	0.85	0.66–1.10	0.227
**eGFR** , (mL/minute/1.73m ^2^ )	≥ 60	1.00	Reference	
	45–59	1.38	1.05–1.82	0.022
	< 45	2.17	1.69–2.79	< 0.001

Abbreviations: BMI, body mass index; eGFR, estimated glomerular filtration rate; NIHSS, National Institutes of Health Stroke Scale; TIA, transient ischemic attack.

## DISCUSSION

In the current study, involving a large prospective cohort, we analyzed the influence of hospital type (public versus private) on mortality and survival after stroke. There was no difference in terms of 30-day survival and risk of death between stroke patients admitted to a public hospital with a dedicated stroke unit and those admitted to two private hospitals in the same city after adjustment for potential confounders.

The characteristics of the stroke population herein studied are similar to those of the samples of other studies conducted in Brazil regarding type of stroke and clinical and sociodemographic profiles. 12 13 14 15 Thus, a higher incidence in men in the older age group, with ischemic stroke being the most common type, was also found in the present study. Regarding the phenotype of stroke patients admitted to the public hospital, a lower proportion of patients aged over 65 years was found, but with a higher prevalence of diabetes, hypertension, and severity of stroke compared with those admitted to the two private hospitals. A previous study 16 compared the clinical outcomes and characteristics of patients admitted to 27 public and private hospitals in the city of Ribeirão Preto, state of São Paulo; although the public hospital patients were not as elderly, they also presented more comorbidities. 16 Similarly, another study 6 compared the clinical outcomes of stroke patients admitted to two reference centers (one public and one private) for stroke in the city of Porto Alegre, Southern Brazil. Both hospitals had stroke care units and followed similar care protocols. In terms of patient phenotype, the authors 6 also observed a lower mean age among patients admitted to the public hospital, as well as a higher prevalence of diabetes, hypertension, and severity of stroke on admission compared with the private hospital patients. It is known that patients who rely exclusively on the SUS have a lower socioeconomic status than those with private health insurance. 17 Thus, a younger population with less access to control of these risk factors could partly explain these characteristics found in stroke patients treated in the public service. 18 19

With regard to lethality, a study 12 conducted in 3 Brazilian state capitals, similar to the present study, found a 28-day lethality rate for stroke of 12.5% (95%CI: 10.4–14.5%). A large population-based study 14 involving hospitals in 5 Brazilian cities with more than 100 thousand inhabitants and from all regions except the North found a higher 30-day mortality rate for ischemic stroke in hospitals in regions with lower socioeconomic levels (23–37%) compared with those in regions with higher levels (10–13%). The study 14 included both public and private hospitals, but did not evaluate specific differences according to the type of hospital; however, the one region with a public hospital with a stroke unit presented a lower mortality rate. In the present study, although there was a trend toward lower mortality among patients admitted to private hospitals, no difference was found between hospital types when gross survival at 30 days was analyzed. A study 13 based on data from the Computer Science Department of the SUS (Departamento de Informática do SUS, DATASUS, in Portuguese) evaluated the trend of hospitalization for stroke and case fatality rate between 2009 and 2016 among more than 1 million hospitalization records selected according to stroke diagnosis codes from the International Statistical Classification of Diseases and Related Health Problems, 10th revision (ICD-10) in public hospitals. The authors 13 found that, although the age-adjusted hospitalization rate and age-adjusted mortality rate decreased during this period, there was a significant increase in mortality among patients aged 70 to 79 and older than 80 years, by 22.3% and 28.7%, respectively. 13 Although advanced age plays an important role in the mortality of these patients, 20 we cannot exclude the hypothesis that the lack of an increase in the number of referral units for acute stroke care in the public network may have contributed to the mortality of these patients. In the aforementioned study 6 conducted in Porto Alegre, there were no differences in mortality between the public and private hospital patients, but those admitted to the public hospital had greater disability 3 months after the event. An observational study 21 also based on DATASUS data compared the mortality among more than 850 thousand patients admitted to public and private hospitals in the states of São Paulo and Rio Grande do Sul, with stroke as the main cause of death. The authors 21 did not adjust for the presence of a stroke unit in the hospitals studied. The probability of death adjusted for the type of hospital funding (public versus private) was of 9% in publicly-funded hospitals compared with 5% in privately-funded hospitals. Among the possible causes associated with higher mortality in patients admitted to public hospitals, the authors 21 point to differences in clinical practice based on best evidence, access to high-cost technologies and procedures, and the size and complexity of the hospital itself. In Brazil, although the Ministry of Health has included thrombolytic therapy for the acute treatment of stroke in public hospitals throughout the country since 2012, 15 many hospitals in more remote regions still do not provide this treatment on an ongoing basis. 22 A meta-analysis 7 of 29 studies examined the impact of dedicated stroke units on acute hospital care. The authors 7 found a higher probability of survival (odds ratio [OR]: 0.78; 95%CI: 0.68–0.91) and physical independence at 1 year among those treated in stroke units. 7 Although the cost per patient treated in specialized units and with the availability of reperfusion therapy may be higher initially, subsequent direct costs (readmissions and rehabilitation) and indirect costs (loss of productivity) can be minimized. 2

The current study has several limitations. First, it was impossible to analyze time-dependent laboratory variables that could impact the outcome studied. Specific data on the onset and results of thrombolytic therapy or thrombectomy were also impossible to consider, but the indications for such procedures followed the recommended clinical criteria. 23 Although both types of hospitals have intensive care units and multidisciplinary teams, it is impossible to avoid the lack of information regarding patients who require intensive care and the number of appointments performed by interdisciplinary teams; such factors could also affect the outcome studied, as pointed out in other studies. 24 25 Moreover, considering that HMSJ is a public referral hospital not only for stroke but also for cancer and trauma, we cannot say that all patients admitted to it stayed in a specific ward for stroke care throughout their stay, but they were managed by a team of neurologists and multidisciplinary care present in the hospital. Another limitation that may have influenced the outcome, especially after hospital discharge, was the lack of information on the socioeconomic status, level of schooling, and postdischarge healthcare network. Since many Brazilians with private health insurance have a higher socioeconomic status and level of schooling, 26 admission to a public hospital could be a proxy measure for factors not directly assessed. Finally, thew current study focused on a single public hospital that established a stroke unit in 1997, approximately 17 years before the ministerial decree promoting the creation of stroke units nationwide. No private hospitals with stroke units were included in the analysis, which is another limitation. Therefore, any extrapolation of the results should be approached with caution.

Despite the limitations, the present study reinforces the potential benefits of stroke units for patients. Considering the structural and resource challenges often faced by public hospitals in Brazil, the presence of stroke units may help to lessen their impact on stroke outcomes, particularly when compared with hospitals with greater resource availability.

The current study, derived from the largest stroke registry in Brazil, emphasized the importance of public policies to optimize stroke victims' care through stroke care units in hospitals with urgent and emergency care. Furthermore, considering the real-world difficulties of public hospitals in Brazil, the implementation of stroke care units can help equate the care of these patients to that offered in private hospitals.
